# Dietary Diversity Predicts the Adequacy of Micronutrient Intake in Pregnant Adolescent Girls and Women in Bangladesh, but Use of the 5-Group Cutoff Poorly Identifies Individuals with Inadequate Intake

**DOI:** 10.1093/jn/nxy045

**Published:** 2018-05-02

**Authors:** Phuong Hong Nguyen, Lieven Huybregts, Tina G Sanghvi, Lan Mai Tran, Edward A Frongillo, Purnima Menon, Marie T Ruel1

**Affiliations:** 1 international Food Policy Research Institute, Washington, DC; 2 FHI 360, Washington, DC; 3 University of South Carolina, Columbia, SC

**Keywords:** adolescent, Bangladesh, pregnant, Minimum Dietary Diversity for Women

## Abstract

**Background:**

The Minimum Dietary Diversity for Women (MDD-W) indicator based on a 10-food group women dietary diversity score (WDDS-10) has been validated to assess dietary quality in nonpregnant women. Little is known about its applicability in pregnant women, and specifically pregnant adolescent girls with higher nutrient requirements.

**Objectives:**

This study aimed to *1*) compare the adequacy of micronutrient intakes between pregnant adolescent girls and women, *2*) examine the performance of WDDS-10 in predicting the mean probability of adequacy (MPA) of 11 micronutrients, and *3*) assess how well the MDD-W cutoff of 5 groups performed in pregnant adolescent girls and women.

**Methods:**

We used data from a 2015 household survey in Bangladesh (*n* = 600). Nutrient intakes were estimated with a multiple-pass 24-h recall and WDDS-10 was assessed through the use of a list-based method. Multiple linear regression models adjusted for geographical clustering assessed the association between WDDS-10 and MPA. Sensitivity and specificity analysis assessed the accuracy of MDD-W in correctly classifying individuals into high (MPA >0.6) or low MPA.

**Results:**

Dietary intakes of pregnant adolescent girls and women were similar in energy intake, WDDS-10 (5.1 ± 1.4), MPA (0.40 ± 0.12), and micronutrient intakes. Probabilities of adequacy were ~0.30 for riboflavin, vitamin B-12, calcium, and zinc; 0.12–0.15 for folate; 0.16–0.19 for vitamin A; and extremely low for iron at 0.01. The WDDS-10 was significantly associated with MPA in both groups and predicted MPA equally well at population level (SD of residuals 0.11 for both). Use of the 5-food groups cutoff for MDD-W to classify individuals’ diets into MPA >0.6, however, resulted in a low correct classification (~40%). A cutoff of 6 food groups markedly improved correct classification.

**Conclusions:**

The WDDS-10 predicted MPA equally well for pregnant adolescent girls and women at population level. The MDD-W indicator performed poorly in classifying individuals with MPA >0.6. *J Nutr* 2018;148:790–797

## Introduction

Micronutrient deficiencies are prevalent among women of reproductive age in developing countries (1), posing several adverse effects on the health, function, and survival of women. These deficiencies are exacerbated during pregnancy when nutrient requirements increase to support maternal physiologic changes and fetal growth, and, if not addressed appropriately, can lead to poor pregnancy outcomes and increased risk of maternal and perinatal mortality Prenatal micronutrient deficiencies among adolescent girls are even more likely due to higher nutritional requirements during the growing and maturing process of the adolescent mother (2, 3).

Among several causes of micronutrient deficiencies, insufficient intake and poor bioavailability of micronutrients are the key concerns (4). Results from a systematic review of 62 studies in developing countries in Africa, Asia, and Latin America and the Caribbean show that imbalanced macronutrient and inadequate micronutrient intakes are common in pregnant women because of their generally monotonous diets, which are predominantly based on plants or cereals and lack micronutrient-dense foods such as vegetables, fruits, and animal-source foods (5). Among pregnant adolescent girls, a review of 9 studies in developed countries found that the average intakes of energy, fiber, and several key micronutrients were below recommended levels (6). Information on micronutrient intake during pregnancy among adolescent girls from low- and middle-income countries, however, is scant, and particularly so in South Asia.

The guideline on Minimum Dietary Diversity for Women (MDD-W) was developed and released by the FAO of the United Nations and FHI 360 in 2016; it proposes a simple dichotomous indicator to assess the dietary quality of women of reproductive age at the population level (7). Earlier research-based rec-ommendations published in 2011 suggested the use of 9 food groups for the Women’s Dietary Diversity Score, but did not propose a dichotomous indicator (4).The 2016 guideline, which was derived from updated research through the use of additional datasets, proposed the MDD-W indicator based on a 10-food group Women’s Dietary Diversity Score, which we will refer to as WDDS-10 (8). The guideline recommends use of consumption of ≥5 out of the 10 food groups as an indicator of a high probability of micronutrient adequacy of women’s diets for 11 micronutrients. Women who consume food items from ≥5 of the 10 food groups are considered more likely to consume foods from animal sources, pulses, nuts or seeds, and fruits or vegetables and to have a greater probability of micronutrient adequacy than those who consume ≤4 food groups (8). The ability of MDD-W to classify women into categories of high and low micronutrient adequacy was evaluated through the use of consumption data from nonpregnant and nonlactating women of reproductive age (8, 9), but has not yet been evaluated in samples of pregnant women, and more specifically in pregnant adolescent girls who have higher nutrient requirements (10). Therefore, it is unknown whether the cutoff of 5 out of 10 food groups is associated with a higher mean probability of adequacy (MPA) for essential micronutrients in pregnant adolescent girls and women compared to those who consume ≤4 food groups.

This study used data from Bangladesh to compare the food and nutrient intakes and micronutrient adequacies of pregnant adolescent girls and women with the use of the probability of adequacy approach. We also examined the performance of the WDDS-10 in predicting MPA and assessed how well the MDD-W cutoff of 5 food groups performs in pregnant adolescent girls and women.

## Methods

### Data source and study population

We used baseline data collected between June and August 2015 as part of a study which assessed the feasibility of integrating a package of maternal nutrition interventions in a Maternal, Neonatal and Child Health program implemented by a large nongovernmental organization (BRAC) in Bangladesh. The survey was carried out in 20 subdistricts (*upazilas*) from 4 districts (Mymensingh, Rangpur, Kurigram, and Lalmonirhat) in northern Bangladesh. Within each subdistrict, 5 unions and 2 villages within each union were randomly selected to yield a total sample of 200 villages. Within each village, a household census was conducted to create a list of pregnant women. From these census lists, pregnant women were selected for the survey through the use of systematic sampling beginning with a random seed start point. The desired sample size was 30 households/village, yielding a total of 600 pregnant women.

The study received ethical approval from the Institutional Review Boards of BRAC University in Bangladesh and the International Food Policy Research Institute, Washington, DC. Written informed consent was obtained from all women aged ≥18 y. For girls <18 y of age, we obtained their assent and the permission of their guardians, i.e., their parents or husbands, to participate in the study.

### WDDS-10

Intake of different food groups during the 24 h prior to the survey was collected with the use of the list-based method (7), in which the enumerator read a list of foods and beverages from each group to the respondent, and asked her if she had consumed them during the previous day and night. We used the 10-food group score as proposed by the MDD-W guideline (7) that consists of: *1*) starchy staple foods, *2*) beans and peas; *3*) nuts and seeds; *4*) dairy products (milk, yogurt, and cheese); *5*) flesh foods (meat, fish, poultry, and liver or organ meats); *6*) eggs; *7*) dark green vegetables; *8*) vitamin A-rich fruits and vegetables; *9*) other vegetables; and *10*) other fruits. Each group was assigned a score of 1 if consumed and 0 if not consumed. The WDDS-10 was the sum of the 10 categorized food groups and thus ranged from 0 to 10. We also generated the WDDS-10 using multiple-pass 24-h dietary intake data to explore if the data collection method would alter our conclusions.

### Assessment of food and nutrient intakes

Food intake was assessed through the use of 1 multiple-pass 24-h diet recall conducted by an enumerator team specially trained for this purpose. Women were asked to describe all foods and beverages they had consumed during the preceding 24 h, including time of consumption, sources where the food was obtained, cooking method, and portion size. Several visual aids (standard pots, plates, bowls, cups, spoons, and other common household utensils) were used to help the women estimate the portion sizes consumed. Recipes of composite dishes were recorded by asking the women who prepared the composite dishes to show the raw food ingredients used. The amounts of each ingredient used were then measured with the use of an electronic dietary scale with a precision of 2 g. If the ingredients were not available at home, the enumerator requested to see the amounts of ingredients used in the recipe by borrowing from a neighbor’s house. If the respondent was unable or unwilling to show (or borrow) the ingredients, the enumerator asked about the size of items and how many or how much was cooked. The enumerator then estimated the weight from a standard list developed by buying all possible ingredients and measuring them at the office during training and prior to survey commencement.

The Bangladeshi food composition table (11) and appropriate conversion factors were used to calculate intakes of energy, macronutrients (protein, fat, and carbohydrate), and micronutrients (iron, calcium, zinc, vitamin A, thiamin, riboflavin, niacin, vitamin B-6, vitamin B-12, vitamin C, and folate). Estimated energy requirements (EER) were calculated for each individual through the use of the equations for basal metabolic rate (estimated from the individual’s age, gender, and weight), level of physical activity, and pregnancy status (12). We used for level of physical activity the factors of 1.4 for low, 1.7 for moderate, and 2.0 for high (13), assigned based on the main occupation reported. Because extra energy intake is required to support adequate gestational weight gain and increases in basal metabolic rate, we added an additional energy requirement for pregnancy of 350 kcal/d for the second trimester and 500 kcal/d for the third trimester of pregnancy (14).

### Usual intakes, micronutrient requirements, and probability of adequacy

We used the Estimated Average Requirements (EAR) and CVs as proposed by the WHO (12) to calculate the probability of adequacy for each micronutrient. The EARs for iron were adjusted to 10% bioavailability (12). The EAR used for zinc was for refined diets as defined by the International Zinc Consultative Group because refined cereal-based diets are typical in Bangladesh (15). Because no EAR is available for calcium, we compared intake levels to the Adequate Intake using the method proposed by Foote et al. (16).

The usual intake distributions for micronutrient intakes were generated with the use of a measurement-error model based on a simple linear regression adjusting for the intraperson or day-to-day variance of the observed nutrient intakes (17). Because this survey only conducted one 24-h recall, we used intraperson variance from a similar dietary intake survey with 2 nonconsecutive 24-h recalls conducted in the same study districts among pregnant adolescent girls and women (18). We applied a Box-Cox transformation to the skewed nutrient intake distribution of every micronutrient to obtain symmetrical distributions. For each participant and micronutrient, we calculated the best linear unbiased predictor of the individual’s usual intake (17) which was then used to calculate the probability of adequacy for every nutrient. Probability of adequacy was calculated as the probability that a girl or woman’s usual intake was above the EAR during pregnancy (10). The MPA of intake of micronutrients for each girl or woman was calculated as the mean of the probabilities of adequacy for the 11 micronutrients.

### Other variables

In addition to dietary intake data, we collected data on household assets ownership, housing conditions, household food insecurity, women’s occupation, and education. Women’s height was measured with the use of a length board, and weight was recorded with the use of an electronic scale and a standard method (19) by trained and standardized field staff. Women’s BMI was calculated as kg/m^2^. A principal component analysis was applied to create an index for household socioeconomic status based on data on housing conditions and as-set holdings. The first principal component that explained most of the variance was divided into tertiles to indicate household socioeconomic status (20, 21). Household food insecurity was measured and calculated through the use of FANTA (Food and Nutrition Technical Assistance)/USAID (United States Agency for International Development)’s Household Food Insecurity Access Scale (22).

### Data analysis

Characteristics of pregnant adolescent girls and women were compared through the use of a Student’s *t* test for continuous variables or chi-square test for categorical variables. Because most distributions of nutrient intakes were skewed, we reported and compared median intakes and IQRs of food group and nutrient intakes for pregnant adolescent girls and women, using nonparametric Wilcoxon’s Rank Sum tests.

Multivariable regression models were used to examine the association between WDDS-10 and MPA, with models either adjusted or not adjusted for energy intake, correcting standard errors for geographical clustering by subdistricts with a Huber-White sandwich estimator (23). We used AUC values from receiver operating characteristic analysis to assess the accuracy of the MDD-W indicator in predicting diets of pregnant adolescent girls and women with MPA >0.6. The value of 0.6 was selected to ensure comparability with the multicountry analysis used to validate the MDD-W among nonpregnant women (24). We estimated the indicator’s sensitivity, specificity, degree of correct classification, and positive predictive value, and used these characteristics to assess the performance of the MDD-W indicator using the 5 food groups cutoff to correctly identify pregnant adolescent girls and women with MPA > 0.6. Finally, we repeated our analysis using a WDDS-10 generated from the multiple-pass 24-h dietary recall data to assess if the data source for the WDDS-10 affected the study results. Statistical significance was set at 5% and all tests were 2-sided. Analyses were done through the use of Stata 14.2 (Statacorp, College Station, TX).

## Results

### Description of the samples

More than one-quarter of the sample were pregnant adolescent girls with a mean age of 17.5 y (range: 13–19 y). Among pregnant adolescent girls, 59% were aged 18–19 y, 40% were aged 15–17 y, and 1.2% were younger than 15 y of age. The mean age of pregnant women was 26 y (range: 20–43 y) with three-quarters of them being <30 y old. Compared to women, a higher percentage of adolescent girls had attended school, and on average, adolescent girls had completed more years of schooling (Table 1). In both groups, >80% did not complete high school. About 90% of the sample reported housework or being housewives as their main occupation with no difference between groups. Compared to women, adolescent girls had similar height (150 cm), but lower body weight (48 compared to 51 kg) and lower BMI (21.3 compared to 22.5). No differences were observed in household socioeconomic status between the 2 groups, but household food insecurity was slightly lower in the adolescent group.

**Table 1:** Sample characteristics^1^

Characteristics	Pregnant adolescent girls (n = 162)	Pregnant women (n = 438)
Age, y	17.5 ± 1.20***	26.3 ± 4.62
Education level, %		
Never attended school	3.09***	15.5
Primary school (grade 1–5)	25.3	33.8
Middle school (grade 6–9)	54.3	41.1
High school or higher	17.3	9.59
Occupation, %		
Household work/housewife	92.0*	87.0
Self-employment	6.17	9.13
Other	1.85	3.88
Obstetric history		
Age when first got married, y	16.0 ± 1.41**	16.4 ± 2.71
Age having first child, y	17.3 ± 1.26***	19.0 ± 3.07
Number of times have been pregnant, y	1.16 ± 0.40***	2.77 ± 1.27
Number of living children,%		
0	91.4***	16.4
1	8.64	43.2
>2	0.00	40.4
Gestational age, %		
Second trimester	48.8	45.5
Third trimester	51.2	54.5
Maternal anthropometry		
Height, cm	150 ± 5.56	151 ± 5.59
Weight, kg	48.2 ± 6.59***	51.0 ± 8.36
BMI, kg/m^2^	21.3 ± 2.41**	22.5 ± 3.24
Household characteristics		
Household size, n	4.13 ± 1.98	4.05 ± 1.62
Household social economic status,^2^ %		
Low	29.0	34.9
Middle	34.0	33.1
High	37.0	32.0
Household food insecurity,^3^ %	37.0*	42.5

1 Values are means ± SDs or percentages. *’**’***Signfiicantly different: *P < 0.05, **P < 0.01, ***P < 0.001.

2 A household economic status index was constructed via principal components analysis with variables on ownerships and assets. It is a standardized score with mean = 0 and SD = 1, divided into tertiles.

3 Household food security was measured through the use of FANTA (Food and Nutrition Technical Assistance)/USAID (United States Agency for International Development)’s Household Food Insecurity Access Scale (22); the scale was then divided into food-secure and food-insecure groups (including mild, moderate, and severely insecure).

### Dietary intake patterns

There were no statistically significant differences between pregnant adolescent girls and women in the amount of food consumed in the previous day for most food groups, except beans and flesh foods which was slightly higher in the adolescent group (Table 2). Over 80% of our sample had consumed animal-source foods in the previous 24 h, but the median intake was low (55–75 g). By contrast, the overall median intake of rice and starchy staple foods was 550 g (raw weight). Whereas all women consumed starchy staple foods and >90% consumed other vegetables, only about one-third consuemd dairy and beans or peas, one-quarter consumed eggs and vitamin A-rich fruit and vegetables, and <4% consumed nuts or seeds. The mean WDDS-10 was 5.1 ± 1.4 food groups, and 65% of women consumed ≥5 food groups. Use of the 24-h recall data to compile the food group score resulted in a slightly higher WDDS-10 compared to the WDDS-10 produced by the list-based method (5.9 compared to 5.3 food groups in pregnant adolescent girls, and 5.6 compared to 5.0 for pregnant women). On average, 40.3% of pregnant women consumed iron folic acid tablets the day before the survey.

**Table 2:** BIntake of food groups among pregnant adolescent girls and women^1^

	Proportion consumed food groups,^2^ %		Quantity of food intake,^3^ g/d	
	Pregnant adolescent girls (n = 162)	Pregnant women (n = 438)	Pregnant adolescent girls (n = 162)	Pregnant women (n = 438)
All starchy staple foods	100	100	557(464, 693)	540(420, 670)
Beans and peas	39.5	34.9	79.4* (36.9,146)	62.3(27.0,118)
Nuts and seeds	3.7	2.5	0.00 (0.00, 0.00)	0.00 (0.00,0.00)
Dairy	42.6	35.4	0.00 (0.00, 200)	0.00 (0.00,125)
Flesh foods	87.0	83.1	75.0* (20.0,170)	55.0(12.5,120)
Eggs	30.3	23.7	0.00 (0.00, 25.26)	0.00 (0.00, 0.00)
Dark green vegetables	45.1	48.0	67.0(7.75, 206)	60.5(8.85,194)
Vitamin A-rich fruits and vegetables	25.9	23.7	0.00 (0.00,0.00)	0.00 (0.00,0.00)
Other vegetables	92.0	91.3	111 (69.5,200)	103(53.4,176)
Other fruits	61.1	56.4	96.0(0.00,210)	83.5(0.00,240)
WDDS-10	5.27 ± 1.51	4.99 ± 1.37	—	—
Proportion consumed ≥5 food groups	66.7	63.7	—	—

1 Values are medians (IQRs), means ± SDs, or percentages. *Significantly different, *P* < 0.05. WDDS-10: Women Dietary Diversity Score based on 10 food groups.

2 Data for food group consumption and dietary diversity score were from the short form list-based method.

3 Data from 24-h recall.

### Energy and macronutrient intakes

There were no statistically significant differences between pregnant adolescent girls and women in energy and macronutrient intakes in the previous day. Overall, the mean energy intake was~2330 kcal/d with 75% of energy from carbohydrate, 11% from protein, and 14% from fat (Table 3).

**Table 3:** Mean energy and macronutrient intake among pregnant adolescent girls and women^1^

Dietary intake	Pregnant adolescent girls (n = 162)	Pregnant women (n = 438)
Energy, kcal/d	2387 ± 795	2311 ± 831
EER,^2^ kcal/d	2142 ± 151	2125 ± 158
Energy intake <85% of EER, %	25.9	28.8
Total carbohydrate, g/d	457 ± 162	444 ± 160
Total fat, g/d	39.5 ± 25.3	38.2 ± 31.3
Total protein, g/d	68.6 ± 26.3	65.6 ± 26.8
Energy from carbohydrate, %	74.8	75.0
Energy from fat, %	14.2	14.0
Energy from protein, %	11.0	11.0

1 Values are means ± SDs or percentages. EER, Estimated Energy Requirement.

2 Values are means ± SDs or percentages. EER, Estimated Energy Requirement.EER was calculated for each individual through the use of the equations for basal metabolic rate (estimated from the individual’s age, gender, and weight), level of physical activity (12), and added pregnancy energy deposition of 340 kcal/d for the second trimester and 452 kcal/d for the third trimester of pregnancy.

### Micronutrient adequacy

There were no statistically significant differences in median micronutrient intakes between pregnant adolescent girls and women (Table 4). Median intakes were below the EAR for 7 micronutrients, but not for thiamin, niacin, vitamin B-6, and vitamin C. Probabilities of adequacy were ~0.30 for riboflavin, vitamin B-12, calcium, and zinc; ~0.16–0.20 for vitamin A; 0.12–0.15 for folate; and extremely low for iron at 0.01. There were no statistically significant differences between pregnant adolescent girls and women in mean MPA (0.40 ± 0.12) or probability of adequacy for individual micronutrients except for calcium (which was lower in adolescent pregnant girls) and zinc (which was higher). The proportion of women with MPA above the cutoff >0.6 was low at 5.8%.

**Table 4:** Selected vitamin and mineral intake from diet among pregnant adolescent girls and women^1^

			Quantity of micronutrient intake		Probability of adequacy	
	Requirement		Pregnant adolescent girls	Pregnant women	Pregnant adolescent girls	Pregnant women
	EAR^2^	SD	*n* = 162	*n* = 438	*n* = 162	*n* = 438
Vitamin A (RAE), µg	370	74	177 (56.4, 388)	191 (75.6,507)	0.16 ± 0.27	0.20 ± 0.30
Thiamin, mg	1.2	0.12	1.67(1.24,2.07)	1.52(1.20,1.95)	0.64 ± 0.22	0.62 ± 0.23
Riboflavin, mg	1.2	0.12	0.83 (0.50,1.29)	0.79(0.49,1.19)	0.34 ± 0.33	0.36 ± 0.32
Niacin, mg	14	2.1	29.5 (23.2, 36.0)	28.2(22.4,34.4)	0.75 ± 0.01	0.75 ± 0.01
Vitamin B-6, mg	1.6	0.16	3.04 (2.37, 3.69)	2.86 (2.31,3.56)	0.75 ± 0.01	0.75 ± 0.01
Folate, µg	520	52.0	259(128,456)	222(130, 371)	0.15 ± 0.25	0.12 ± 0.22
Vitamin B-12, µg	2.2	0.22	1.28 (0.37, 2.34)	1.09 (0.38, 2.04)	0.32 ± 0.34	0.31 ± 0.33
Vitamin C, mg	46	4.6	165 (46.9,359)	155(51.9, 374)	0.74 ± 0.08	0.75 ± 0.01
Calcium, g	800	100	241 (91.5, 523)	274 (120, 495)	0.26 ± 0.21	0.34 ± 0.22
Iron,3 mg	24.9	2.34	9.47(6.47,13.4)	8.79(6.50,12.1)	0.01 ± 0.01	0.01 ± 0.02
Zinc,^4^ mg	8	1.0	7.59(5.54,10.4)	7.02(5.39,9.03)	0.30 ± 0.29	0.25 ± 0.27
MPA	—	—	—	—	0.40 ± 0.13	0.41 ± 0.11

1 Values are medians (IQRs) or means ± SDs. ****Significantly different: *P < 0.05, ***P < 0.001. EAR, estimated average requirement; MPA, mean probability of adequacy; RAE, retinol activity equivalent.

2 EARs are based on the WHO/FAO recommendations for pregnant women (25).

3 3Iron: assuming 10% bioavailability.

4 4Zinc was for refined diets as defined by the International Zinc Nutrition Consultative Group (15).

### Association between MDD-W score and MPA

For both pregnant adolescent girls and women, WDDS-10 was positively associated with MPA (Table 5). The unadjusted models explained 34% and 16% of the variance for adolescent girls and women, respectively, and the SD of the residuals was 0.11 for both. Pregnant adolescent girls who consumed 5, 6, 7, and 810 food groups had 0.06, 0.15, 0.16, and 0.22 higher MPA, compared to those who consumed ≤4 food groups. Adjusting the model for energy attenuated the magnitude of effects, but all remained statistically significant. Similar findings were observed for pregnant women, with coefficients ranging from 0.06 to 0.15 for the non-energy-adjusted model and from 0.05 to 0.12 for the energy-adjusted model.

**Table 5:** Multivariable regression of determinants of mean probability of adequacy among pregnant adolescent girls and women^1^

	Pregnant adolescent girls		Pregnant women	
	Not adjusted for energy (n = 162)	Adjusted for energy (n = 162)	Not adjusted for energy (n = 438)	Adjusted for energy (n = 438)
MDD-W score^2^				
2–4	Ref.	Ref.	Ref.	Ref.
5	0.06*	0.03	0.06***	0.05***
6	0.15***	0.09***	0.08***	0.06**
7	0.16***	0.11**	0.12***	0.09***
8–10	0.22***	0.14**	0.15***	0.12***
Energy intake, kcal/d	—	0.0001**	—	0.0001***
Constant	0.35	0.23	0.36	0.23
Adjusted R^2^	0.34	0.46	0.16	0.36
SD of residuals	0.11	0.10	0.11	0.09

1 Values are coefficients. *,**,***Significantly different: *P < 0.05, **P < 0.01, ***P < 0.001. MDD-W:Minimum Dietary Diversity for Women; Ref., reference.

2 Data for the MDD-W score were from the short form list-based method.

### Indicator performance and identification of cutoffs

We used AUC values to assess the accuracy of the MDD-W indicator in predicting an MPA ≥0.6. The AUC was 0.76 (95% CI: 0.63, 0.90) for pregnant adolescent girls and 0.78 (95% CI: 0.70, 0.87) for women (Table 6). These values differed significantly from the null value (0.5). With the use of an MPA cutoff of 0.6, the cutoff of 5 food groups gave high sensitivity (>90% for both groups), but low specificity (35% for adolescent girls and 38% for women). That is, the cutoff of ≥5 correctly identified nearly all pregnant adolescent girls and women who had MPA >0.6, but resulted in low specificity (percentage of women correctly identified as having a low MPA). Furthermore, this cutoff resulted in low positive predictive value (i.e., percentage of adolescent girls and women classified as high MPA who actually had a high MPA) and a low percentage of correctly classified adolescent girls and women (~40%).

**Table 6:** Accuracy of indicators for classifying mean probability of adequacy >0.60 for pregnant adolescent girls and women^1^

Food group cutoff^2^	Pregnant adolescent girls (*n* =162)				Pregnant women (*n* = 438)			
	Sensitivity	Specificity	Total correctly classified	Positive predictive value	Sensitivity	Specificity	Total correctly classified	Positive predictive value
≥1	—	—	—	—	—	—	—	—
≥2	100	0.00	7.41	7.41	100	0.24	5.48	5.26
≥3	100	1.33	8.64	7.50	100	1.93	7.08	5.35
≥4	100	12.7	19.1	8.39	100	13.7	18.3	6.04
≥5	91.7	35.3	39.5	10.2	95.5	37.8	40.6	7.53
≥6	83.3	61.3	63.0	14.7	78.3	72.1	72.4	13.4
>7	50.0	80.7	78.4	17.1	47.8	87.0	84.9	16.9
≥8	33.3	93.3	88.9	28.6	17.4	96.1	92.0	20.0
≥9	0.00	99.3	92.0	0.00	0.00	99.5	94.3	0.00
≥10	0.00	99.3	92.6	0.00	0.00	100	94.8	—
AUC	0.76(95% CI: 0.63, 0.90)				0.78 (95% CI: 0.70, 0.87)			

1 T1Values are percentages.

2 Data for the 10-food group women dietary diversity score were from the short form list-based method.

A cutoff of 6 food groups performed better in this population in correctly identifying women who had high (sensitivity: 78–83%) or low MPA (specificity: 61–72%), with a higher percentage of correctly classified pregnant adolescent girls (63%) and women (72%) than with a cutoff of 5 food groups. Positive predictive values remained low (13–15%), however, even with a cutoff of 6 food groups. Overall, the optimal cutoff values for MDD-W in this population did not differ by whether they were adolescent girls or women. Use of the WDDS-10 derived from the 24-h dietary recall data generated similar results with a low correct classification (25–27%) and poor positive predictive values (<10%) (results not shown).

## Discussion

Both pregnant adolescent girls and women in our sample from Bangladesh had imbalanced macronutrient intake (with high carbohydrate and low fat and protein intakes) and inadequate micronutrient intake (with particularly low probability of adequacy for key micronutrients such as iron, vitamin A, and folate). Although mean dietary diversity measured by the WDDS-10 was >5 out of 10 food groups in both pregnant adolescent girls and women, only 5.8% of them had an MPA >0.6 for the 11 micronutrients analyzed. The WDDS-10 was significantly associated with MPA and predicted MPA equally well at population level for both adolescent girls and women. The proposed MDD-W cutoff of >5 food groups (7), however, was not suitable for pregnant adolescent girls or women in our sample, possibly due to their higher nutrient requirements compared to nonpregnant women. For our sample, a cutoff of >6 food groups more accurately identified adolescent girls and women with MPA >0.6 as shown by the greater balance achieved in sensitivity and specificity and percentage correctly classified at that cutoff.

Dietary intakes in Bangladesh have been reported in only a few studies, 2 of which were included in the multicountry analyses that were used to generate the 9-group Women’s Dietary Diversity Score and the MDD-W in nonpregnant women of reproductive age (9, 24). A recent analysis of a nationally representative survey, the Bangladesh Integrated Household Survey (26), compared diets and nutrient intakes of nonpregnant adolescent girls and women, and similar to our findings, showed no consistent differences in diets and nutrient intakes between the 2 groups; intakes of iron, vitamin A, and zinc were alarmingly low (27).

The heavy reliance on rice as a main source of energy in the diets of poor Bangladeshi women and children is well-recognized and has been associated with low dietary diversity and grossly inadequate intakes of many micronutrients (28). In our sample, staple foods—rice and other staples—constituted 75% of the total energy intake, confirming the limited potential contribution of nutritious foods from food groups other than staples. High food prices and low incomes have been identified as key barriers that prevent pregnant women from accessing more nutritious foods such as animal-source foods and fresh fruits and vegetables to complement the staple diet (29). Our sample of pregnant adolescent girls and women, however, had a relatively high dietary diversity (mean 5.0–5.3) and 65% achieved the recommended cutoff of 5 food groups. Despite this, the diets of pregnant adolescent girls and women in our sample were highly deficient in several key micronutrients, suggesting that although they may have consumed foods from different groups, the amounts consumed are likely to have been too small to help them achieve overall adequacy in micronutrient intakes. This hypothesis was confirmed in our analysis, which showed that although a high percentage of our sample consumed flesh foods (83–87%), median intake was low (55–75 g). Similar results were found for dark green vegetables, which were consumed by 45–48% of women, but in small amounts (median 60–67 g).

These findings may explain, at least in part, why the MDD-W cutoff of ≥5 food groups did not perform well in our study and resulted in a large proportion of misclassified women (~60%). The specificity of the indicator (i.e., its ability to correctly identify individuals as having a low MPA) was particularly poor, and resulted in a large proportion of pregnant adolescent girls and women being incorrectly classified as having a high probability of adequate micronutrient intake. For our samples of pregnant adolescent girls and women, we obtained better classification (63–72% correctly classified) and a more balanced sensitivity and specificity when applying a cutoff of ≥6 food groups.

The guideline on MDD-W proposes 2 ways to collect the data for WDDS-10 (7) and highlights the advantages and disadvantages of each method. In the main analyses presented in this paper, we used the list-based method to avoid the concern that both WDDS-10 and MPA of micronutrient intake come from the same 24-h recall. Repeating the analyses with the use of a WDDS-10 derived from the 24-h dietary intake data did not change our finding that the MDD-W performed poorly in identifying individuals with MPA >0.6 among pregnant girls and women. The recall of food group intake through the use of the list-based method was done on the same day as the multiple-pass 24-h recall. This had an advantage in that the WDDS-10 derived by the list-based method was similar to the data of the 24-h recall. The downside of conducting 2 recalls on the same day, albeit with a different format and independent enumerator teams, is that the first method might have influenced the performance of the second method.

One limitation of our study is that only one 24-h recall per woman was collected to estimate dietary intakes. Although this is appropriate to measure mean intakes of populations, it is inadequate to capture the day-to-day variation in intakes which inflates the population distribution of intakes and results in in-accurate estimates of population-level intake adequacy. To overcome this limitation, we used external estimates of variation of nutrient intakes from a survey in pregnant women in the same nonintervention community in Bangladesh (18) to estimate the usual nutrient intake of our study population, a method that has been evaluated in other populations (30).

In conclusion, we found no meaningful differences in the dietary intake of Bangladeshi pregnant adolescent girls or women. Both groups had poor diets, with imbalanced macronutrients and alarmingly low intakes of some key micronutrients. These findings raise a great concern because of the possible negative impacts these poor diets may have on maternal health and pregnancy and birth outcomes (31). The findings also highlight the need for antenatal care services to incorporate nutrition interventions that address both problems of food insecurity and limited knowledge regarding healthy diets during pregnancy, as recommended in the new WHO antenatal care guidelines for a positive pregnancy experience (32). The WDDS-10 was found to be useful in predicting MPA in both pregnant adolescent girls and women, but the recommended cutoff of ≥5 food groups used for the MDD-W indicator performed poorly in differentiating women with adequate or low MPA. Our data suggest that in this population of pregnant adolescent girls and women in Bangladesh, a cutoff of ≥6 may perform better. Further studies in other settings are needed to confirm the accuracy of the currently proposed cutoff of 5 food groups for the MDD-W indicator when used to assess dietary diversity among pregnant adolescent girls or women who have higher nutrient requirements than the groups of nonpregnant and nonlactating women that were used to derive the current MDD-W indicator.
